# Exploring fMRI Results Space: 31 Variants of an fMRI Analysis in AFNI, FSL, and SPM

**DOI:** 10.3389/fninf.2016.00024

**Published:** 2016-07-05

**Authors:** Ruth Pauli, Alexander Bowring, Richard Reynolds, Gang Chen, Thomas E. Nichols, Camille Maumet

**Affiliations:** ^1^Warwick Manufacturing Group, University of WarwickCoventry, UK; ^2^Scientific and Statistical Computing Core, National Institute of Mental Health, National Institutes of HealthBethesda, MD, USA; ^3^Department of Statistics, University of WarwickCoventry, UK

**Keywords:** data sharing, functional MRI, provenance, fMRI analysis, neuroimaging

## Background

Data sharing is becoming a priority in functional Magnetic Resonance Imaging (fMRI) research, but the lack of a standard format for shared data is an obstacle (Poline et al., 2012; Poldrack and Gorgolewski, 2014). This is especially true for information about data provenance, including auxiliary information such as participant characteristics and task descriptions. The three most commonly used analysis software packages [AFNI^1^ (Cox, 1996), FSL^2^ (Jenkinson et al., 2012), and SPM^3^ (Penny et al., 2011)] broadly conduct the same analysis, but differ in how fundamental concepts are described, and have a myriad of differences in the pre-processing and modeling steps. The practical consequence is that sharing analyzed data is further complicated by the idiosyncrasies of the particular software used.

The Neuroimaging Data Model [NIDM^4^ (Keator et al., 2013; Maumet et al., 2016)] is an initiative from the International Neuroinformatics Coordinating Facility (INCF^5^) that addresses these practical barriers through the development of a standard format for neuroimaging data. Ultimately, NIDM will provide a standard format that can handle data that has been processed in any of the common software packages. In order to achieve this, the development of NIDM requires publicly available derived data that covers all the major use cases in the main software programs.

The purpose of the current work was to produce a set of results of mass univariate fMRI analyses using the most common software packages: AFNI, FSL, and SPM [which between them cover 80% of published fMRI analyses (Carp, 2012)], utilizing publicly available data from OpenfMRI^6^ (Poldrack et al., 2013). The analyses (‘variants’) presented in this paper cover the most common options available in each software package at each analysis stage, from different Hemodynamic response function (HRF) basis functions through to group-level tests. The tests are arranged so that readers can compare the closest equivalent variants across software packages. In particular, these tests will be useful for comparing the results from default test settings across software packages.

While this collection of analyses was chosen for their relevance to the NIDM project, it also addresses a gap in the literature where publicly available processed data is concerned. Specifically, while there are published comparisons of different processing pipelines, the data are not publicly available (Carp, 2012) or are for resting state fMRI only (Bellec et al., 2016). Others have shared raw data but lack analysis results (Hanke et al., 2014) or do not include comparisons across multiple software packages [e.g., analyses in the The Human Connectome Project^7^ (Van Essen et al., 2013) are performed with FSL only]. Shared raw data is a useful resource, but we argue that shared processed data is also important, both to provide a basis of cross-software comparisons and to create a benchmark for testing of automated provenance software. The dataset presented in this paper is a contribution toward this omission in the literature.

## Methods

### Data Source

Data were downloaded from OpenfMRI’s BIDS-compliant ds000011 dataset^8^ between 09/02/2016 and 15/02/2016. A full description of the paradigm is in the original paper (Foerde et al., 2006). The first task was a training exercise in which participants counted high tones in a series of high-pitched and low-pitched tones (‘tone counting’ condition), and then selected a number that represented the number of high tones (the ‘tone counting probe’ condition, referred to as ‘probe’ hereafter). We modeled both the tone counting and probe conditions, using tone counting as the effect of interest ([1 0] contrast with implicit baseline). Single-subject tests were conducted with data from subject 01 only, while group-level tests were run with all 14 subjects. Analyses were conducted in AFNI, SPM12, and FSL.

In AFNI, single-subject variants were conducted using the uber_subject.py interface, which generates and runs two scripts: cmd.ap.sub_001 and proc.sub_001. Other variants did not require changing options in the interface, so were run directly from the command line, using a copy of the default cmd.ap.sub_001 script. Scripts for group-level tests were created manually.

For each of the SPM variants, a batch.m file conducting the full analysis (using dependencies across processing steps) was created and run with the Batch Editor GUI.

FSL-specific variants were modeled using FSL’s FMRI Expert Analysis Tool^9^, where a.fsf file for the complete analysis was created using the FEAT GUI.

### Pre-defined Settings

In this section, settings held constant over variants (e.g., drift modeling) are described for each of the packages. These pre-defined settings (including pre-processing) were identical for each variant.

### Pre-processing

As slice-time information was not available for this study, this step was not considered in the pre-processing.

In AFNI, pre-processing was conducted using the default settings in the AFNI uber_subject.py graphical interface. First, the BOLD images were rigidly aligned to the skull-stripped anatomical T1-weighted image using a negative local correlation cost function. Next, the anatomical image was registered to standard space using the AFNI default ‘Colin brain’ (TT_N27+tlrc) Talairach space template (Holmes et al., 1998) with an affine transformation and weighted least squares cost function. Head motion correction was performed by rigid body registration of each BOLD volume to the third volume, also using weighted least squares cost function; all three transformations were concatenated to allow a single resampling with cubic interpolation. In addition, volumes presenting an estimated motion greater than 0.3 mm (as estimated at 85% of the distance to the cortical envelope^10^) compared to the previous scan were censored from the first level regression. The BOLD images were smoothed with a 4 mm Full Width at Half Maximum (FWHM) Gaussian smoothing kernel, and each voxel was scaled to have a mean value of 100 across the run with values larger than 200 truncated to that value.

In FSL, pre-processing was conducted using the Brain Extraction Tool (BET)^11^ and the default options of the FEAT GUI. First, the anatomical image was skull-stripped. To correct for motion, each volume from the BOLD images was first registered rigidly to the middle volume using a normalized correlation cost function and linear interpolation (MCFLIRT^12^ tool). After 6 mm FWHM spatial smoothing and global scaling to set median brain intensity to 10,000, first level fMRI model fitting took place in the subject space. The mean realigned fMRI data was rigidly registered to the brain extracted anatomical image using a correlation ratio cost function, followed by affine registration of the anatomical to MNI space (as defined by the ICBM MNI 152 non-linear 6th generation template image), also with correlation ratio cost function. For group or second level fMRI modeling, the preceding registration parameters were composed to directly resample first level contrast estimates and their variance into standard space with trilinear interpolation.

In SPM, pre-processing was conducted with the Batch Editor GUI. To correct for motion, a two-step rigid body registration procedure was performed with a least squares cost function; each volume from the BOLD images was first registered to the first volume, and then registered to the mean of the aligned images (‘Realign: Estimate & Reslice’ function, cubic spline interpolation). The anatomical T1-weighted image was then rigidly registered to the mean BOLD image with a mutual information cost function (‘Coregister: Estimate’ function, cubic spline interpolation). Segmentation, bias field correction and non-linear registration of the anatomical image to standard space (“unified segmentation”) were then conducted (‘Segment’ function); instead of a simple cost function, this process uses a model incorporating tissue class, bias field and spatial deformations to best fit the T1 image data. The estimated deformation field was then used for warping the realigned BOLD images (cubic spline interpolation) and bias corrected anatomical image to MNI space (as defined by the average image of 549 of the subjects from the IXI dataset^13^, cf. spm_template.man for more details) using spline interpolation (‘Normalise: Write’ function). Finally, the normalized realigned BOLD images were smoothed using a 6 mm FWHM Gaussian smoothing kernel (‘Smooth’ function). Global scaling (1 value for whole 4D dataset) was used to set the mean brain intensity to target value of 100^14^.

### Data Analysis

As specified by the tone counting task, for each software package, the subject-level design matrix included at least two regressors (“tone counting” and “probe”).

By default AFNI adds nine additional regressors in the design matrix: an intercept, two to model slow signal drifts using a second-order polynomial, and six motion regressors (three rotations, three shifts), resulting in a design matrix with 11 columns.

By default SPM adds a discrete cosine transform basis to the linear model to account for drift. The default cutoff of 128 s with this 208 s acquisition allowed three regressors. With an intercept, the model has six regressor parameters, though the drift basis columns are not displayed to the user.

In FSL, slow signal drifts were removed from the data and modeled with a Gaussian-weighted running line smoother with bandwidth parameter 60 s^15^, a reduction from the software default value of 100 s, since this is recommended for event-related designs^16^. The design matrix has only two columns, which are mean centered.

### Variants

Users can specify from a range of options at each stage in the analysis processing pipeline. A one-factor-at-a-time design was used to run tests with these different options. In each of the analysis packages, at each processing stage (Column 1, **Table 1**.) a single variant was labeled as a default (Columns 3–5, **Table 1**.). This default variant was usually the same in each software package, except for software-defined defaults, which were left unchanged (e.g., HRF).

**Table 1:** Folder names for variants in each software package (columns 3–5), for each variant type (columns 1–2).

	Variant name	AFNI	FSL	SPM
Model	First-level regression	**Default** afni_default	**Default** fsl_default	**Default** spm_default
	Second level: 1 sample *t*-test with ordinary least squares	afni_group_ols	fsl_group_ols	spm_group_ols
	Second level: 1 sample *t*-test with weighted least squares	afni_group_wls	fsl_group_wls	spm_group_wls
Hemodynamic response function (HRF)	Gamma difference	afni_hrf_gammadiff	fsl_hrf_gammadiff	**Default** spm_default
	Gamma	**Default** afni_default	**Default** fsl_default	NA
	FIR/TENT Basis Function	afni_hrf_tent	fsl_hrf_fir	spm_hrf_fir
Threshold	voxel-wise uncorrected *p* ≤ 0.001	**Default^∗^** afni_default	**Default^∗^** fsl_default	**Default^∗^** spm_default
	voxel-wise uncorrected *t* ≥ 4	afni_thr_voxelunct4	NA	spm_thr_voxelunct4
	voxel-wise (peak-wise) FWE *p* ≤ 0.05	NA	fsl_thr_voxelfwep05	spm_thr_voxelfwep05
	voxel-wise FDR *p* ≤ 0.05	afni_thr_voxelfdrp05	NA	spm_thr_voxelfdrp05
	Cluster-wise uncorrected *k* ≥ 10, cluster-defining threshold *p* ≤ 0.001	afni_thr_clustunck10	NA	spm_thr_clustunck10
	Cluster-wise FWE *p* ≤ 0.05, cluster-defining threshold *p* ≤ 0.001	afni_thr_clustfwep05	fsl_thr_clustfwep05	spm_thr_clustfwep05
Contrast type	*t*-contrast	**Default^∗∗^** afni_default	**Default^∗∗^** fsl_default	**Default^∗∗^** spm_default
	*f*-contrast	afni_con_f	fsl_con_f	spm_con_f
Cluster connectivity	6-Connected: faces	**Default** afni_default	NA	NA
	18-Connected: faces and edges	afni_clustconn_18	NA	**Default** spm_default
	26-Connected: faces, edges, and corners	afni_clustconn_26	**Default** fsl_default	NA
Hypothesis type	One-tailed test	afni_alt_onesided	**Default** fsl_default	**Default** spm_default
	Two-tailed test	**Default** afni_default	NA	NA

*Default tests are marked in bold in the table. ^∗^For group-level analyses, the default threshold is cluster-wise *p* ≤ 0.05 FWE-corrected. ^∗∗^For analyses using flexible basis functions to model the HRF the default test is an *f*-test with an identity matrix of size equal to the number of basis*.

The variants are presented below. Apart from the aspect that had been explicitly changed for that variant, all other stages of the processing pipeline were kept the same as the default analysis. Using this method, at least one analysis was conducted for each possible variant at every stage of the processing pipeline in AFNI, SPM, and FSL.

## Hemodynamic Response Function

### Gamma Function

The HRF was modeled using a Gamma function.

### Difference of Gamma Functions

The HRF was modeled using the difference of two Gamma functions. This is the default in SPM (SPM’s canonical HRF). In FSL, the Double-Gamma HRF option was used (with a phase 0).

### Flexible Factorial Basis Functions

The HRF was modeled using a finite impulse response (FIR) basis set or a set of TENT functions. In FSL, three basis FIR functions spread over 15 s were defined. In SPM, 10 basis FIR functions spread over 20 s were defined. In AFNI, eight (respectively, seven) TENT functions were defined with a 0 s start and a duration of 12 s (respectively, 14 s) for the tone counting (respectively, the probe) regressor.

## Model Variants

### First-Level Regression

The default analysis for all software packages was a single-subject *t*-test on the tone counting contrast.

### Second Level: 1 Sample *t*-test Estimated with Ordinary Least Squares

A one-sample group *t*-test with ordinary least square estimation was performed on the tone counting contrast over the 14 participants.

### 1 Sample *t*-test Estimated with Weighted Least Squares

A one-sample group *t*-test with weighted least square estimation was performed on the tone counting contrast over the 14 participants.

## Threshold

### Voxel-Wise Uncorrected *p* ≤ 0.001

Results were thresholded with a voxel-wise threshold of *p* ≤ 0.001 uncorrected for multiple comparisons.

### Voxel-Wise *t* ≥ 4

Results were thresholded with a voxel-wise threshold of *t* ≥ 4.

### Voxel-Wise (Peak-Wise) FWE *p* ≤ 0.05

Results were thresholded with a voxel-wise threshold of family-wise error rate *p* ≤ 0.05 with correction for multiple comparisons.

### Voxel-Wise FDR *p* ≤ 0.05

Results were thresholded with a voxel-wise threshold of false discovery rate *p* ≤ 0.05 correction for multiple comparisons.

### Cluster-Wise *k* ≥ 10

Results were thresholded with a cluster-wise threshold of 10 voxels. Clusters were defined using a cluster-forming threshold of *p* ≤ 0.001 uncorrected for multiple comparisons.

### Cluster-Wise FWE *p* ≤ 0.05

Results were thresholded with a cluster-wise threshold of family-wise error rate *p* ≤ 0.05 with correction for multiple comparisons. Clusters were defined using a cluster-forming threshold of *p* ≤ 0.001 uncorrected for multiple comparisons.

## Contrast

### *t*-test

The default analysis for all software packages was a *t*-test on the tone counting contrast.

### *f*-test

An *f*-test on the tone counting contrast was performed.

## Cluster Connectivity

### 6-Connected

Neighboring voxels had faces touching. Under this definition a voxel can have up to six nearest neighbors. This is the default in AFNI.

### 18-Connected

Neighboring voxels were defined as those with faces or edges touching. Under this definition a voxel can have up to 18 nearest neighbours. This is the default in SPM.

### 26-Connected

Neighboring voxels had faces, edges, or corners touching. Under this definition a voxel can have up to 26 nearest neighbors. This is the default in FSL.

## Alternative Hypothesis

### One-Tailed Test

A one-tailed test looking at positive effects was performed. This is the default in SPM and FSL.

### Two-Tailed Test

A two-tailed test looking at positive and negative effects was performed. This is the default in AFNI.

## Results

**FIGURE 1:**
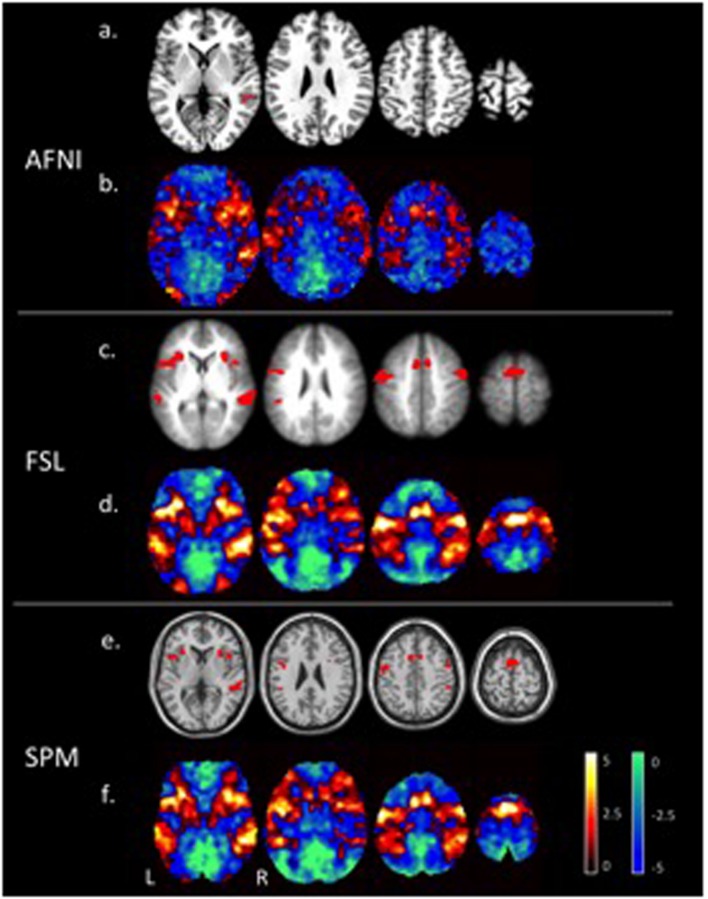
**Areas of significant activations (red) for a one-sided *t*-test at a *p* ≤ 0.05 FWE corrected cluster-wise threshold for the tone counting contrast **(a)**, **(c)**, **(e)**, and un-thresholded *t*-statistic maps (positive values in red to yellow and negative values in blue to green) **(b)**, **(d)**, **(f)**, at the group level are compared for each of the three software packages (AFNI, FSL, and SPM)**.

**Figure 1** shows the tone counting group level results from a one-sided test FWE-corrected *p* ≤ 0.05 cluster-wise inference with a *p* ≤ 0.001 uncorrected cluster forming threshold. Despite differences in smoothing, the unthresholded maps show the same general pattern of activation. Thresholded maps from SPM and FSL (6 mm FWHM smoothing) were most similar, while AFNI (4 mm FWHM smoothing) presented a smaller number of active voxels. Aside from smoothing, an important difference with AFNI is the inclusion of motion regressors in the first level model; this is good statistical practice but can reduce sensitivity if the subject motion is correlated with the regressor of interest.

In comparing software packages, there is naturally a tension between exact matching of parameter sets versus use of recommended defaults. The analyses presented here remain faithful to the default settings where possible. Consequently, the differences between packages can be difficult to interpret fully. We plan future work with carefully matched analyses, in order to further elucidate the differences between software packages using many more datasets.

Finally, it is important to emphasize that these tests are not intended to demonstrate the superiority of any particular software package over the others. Each package has its strengths and weaknesses. For example, SPM cannot compute cluster-size inference by FWE *p*-value, while FSL cannot specify inference by uncorrected cluster threshold. AFNI has immense flexibility in this regard, but the sheer number of options available can make it difficult for the user to judge how to proceed. Ultimately, choice of software will usually be a matter of personal preference for the researcher.

### Data Sharing

The dataset is named fMRI Results Comparison Library and can be available at: http://warwick.ac.uk/tenichols/fmri_results. The folder names for each variant are provided in **Table 1**.

For the AFNI variants, each folder contains scripts for running the analysis (cmd.ap.sub_001 and proc.sub_001 for single-subject tests), scripts for specifying the threshold levels (batch.sh; these are not standard AFNI output, and are included so that future users can run the analysis without manually setting thresholds in the interface), the thresholded dataset (Clust_mask+tlrc) that contains significant clusters, and files that AFNI outputs automatically, saved in the sub_001.results folder.

For the FSL variants, each directory contains the complete FEAT output, which includes the FEAT setup file (design.fsf), motion correction reporting (mc/directory), low-res stats outputs (stats/directory), standard space registration outputs (reg/directory), resampling of stats images into standard space (reg_standard/stats/directory), and time series plots (tsplot/directory). All.html files of the FEAT report are also included.

For the SPM variants, each directory contains a batch.m file to run the analysis, as well as the SPM.mat file containing the design specification. NIFTI files for the regressors, contrasts, and thresholded results are included, and the results report obtained from the analysis has been printed in.pdf format.

Finally, a README.md file is contained in every variant directory, giving a description of the variant and data used in the test.

### Recommended Uses

The dataset includes all the necessary scripts and files for future users to replicate the analyses exactly as they were carried out here. This is especially useful for those seeking quick comparisons between different processing options (both within and between software packages). In AFNI, this also removes the need to enter threshold or cluster information manually via the interface. In addition, the dataset and accompanying information in this paper should be useful for novice neuroimagers seeking clear descriptions and examples of basic tests to guide them in their own research.

## Author Contributions

Analyses were conducted by AB, CM, and RP, with guidance from GC, RR, CM, and TN. The paper was drafted by AB and RP, and written by AB, CM, RP, TN, and RR.

## Conflict of Interest Statement

The authors declare that the research was conducted in the absence of any commercial or financial relationships that could be construed as a potential conflict of interest.

## Footnotes

1 http://afni.nimh.nih.gov/

2 http://fsl.fmrib.ox.ac.uk/fsl

3 http://www.fil.ion.ucl.ac.uk/spm/

4 http://nidm.nidash.org

5 http://www.incf.org

6 https://openfmri.org

7 http://www.humanconnectome.org

8 https://drive.google.com/folderview?id=0B2JWN60ZLkgkMGlUY3B4MXZIZW8&usp=sharing

9 http://fsl.fmrib.ox.ac.uk/fsl/fslwiki/FEAT

10 https://afni.nimh.nih.gov/afni/community/board/read.php?1,149511,149513\#msg-149513

11 http://fsl.fmrib.ox.ac.uk/fsl/fslwiki/BET

12 http://fsl.fmrib.ox.ac.uk/fsl/fslwiki/MCFLIRT

13 http://www.brain-development.org/

14 Due to an over-sized brain mask used for global mean computation, SPM’s intracerebral mean tends to be under-estimated, resulting in a scaled brain mean intensity of 200 or more instead of 100. For more see: http://blogs.warwick.ac.uk/nichols/entry/spm_plot_units/.

15 This parameter is only approximately the FWHM of the smoother’s Gaussian, since FSL uses 2.0 instead of 2.335 in the FWHM-to-sigma conversion. For more see: https://www.jiscmail.ac.uk/cgi-bin/webadmin?A2=ind1109\&L=FSL\&P=R30849

16 http://fsl.fmrib.ox.ac.uk/fsl/fslwiki/FEAT/UserGuide#FEAT_Basics.
